# Effect of *ACTN3* R577X Genotype on Injury Epidemiology in Elite Endurance Runners

**DOI:** 10.3390/genes12010076

**Published:** 2021-01-08

**Authors:** Jorge Gutiérrez-Hellín, Gabriel Baltazar-Martins, Millán Aguilar-Navarro, Carlos Ruiz-Moreno, Jesús Oliván, Juan Del Coso

**Affiliations:** 1 Faculty of Health Sciences, Francisco de Vitoria University, 28223 Pozuelo de Alarcón, Spain; jorge.gutierrez@ufv.es (J.G.-H.); millan.aguilar@ufv.es (M.A.-N.); 2 Exercise Physiology Laboratory, Camilo José Cela University, 28692 Villanueva de la Cañada, Spain; jgsoares@ucjc.edu (G.B.-M.); cruizm@ucjc.edu (C.R.-M.); 3 Faculty of Physical Activity and Sport Sciences, Technical University of Madrid, 28040 Madrid, Spain; jesus.olivan@upm.es; 4 Centre for Sport Studies, Rey Juan Carlos University, 28933 Fuenlabrada, Spain

**Keywords:** athletic performance, exercise-related injury, single nucleotide polymorphism, track and field athlete, α-actinin-3 deficiency

## Abstract

The p.R577X polymorphism (rs1815739) in the *ACTN3* gene causes individuals with the *ACTN3* XX genotype to be deficient in functional α-actinin-3. Previous investigations have found that XX athletes are more prone to suffer non-contact muscle injuries. This investigation aimed to determine the influence of the *ACTN3* R577X polymorphism in the injury epidemiology of elite endurance athletes. Using a cross-sectional experiment, the epidemiology of running-related injuries was recorded for one season in a group of 89 Spanish elite endurance runners. *ACTN3* R577X genotype was obtained for each athlete using genomic DNA samples. From the study sample, 42.7% of athletes had the RR genotype, 39.3% had the RX genotype, and 18.0% had the XX genotype. A total of 96 injuries were recorded in 57 athletes. Injury incidence was higher in RR runners (3.2 injuries/1000 h of running) than in RX (2.0 injuries/1000 h) and XX (2.2 injuries/1000 h; *p* = 0.030) runners. RR runners had a higher proportion of injuries located in the Achilles tendon, RX runners had a higher proportion of injuries located in the knee, and XX runners had a higher proportion of injuries located in the groin (*p* = 0.025). The *ACTN3* genotype did not affect the mode of onset, the severity, or the type of injury. The *ACTN3* genotype slightly affected the injury epidemiology of elite endurance athletes with a higher injury rate in RR athletes and differences in injury location. However, elite *ACTN3* XX endurance runners were not more prone to muscle-type injuries.

## 1. Introduction

The status of elite athlete requires many hours of strenuous training per week that can impose severe physiological and mechanical stress on the human body, ultimately leading to sports injuries. For this reason, injury is an inherent feature of elite sport [1], and injury prevention has become a key aspect of every conditioning program. Because injuries may inhibit regular training, negatively impact sports performance, and even shorten an athlete’s career, the study of all the risk factors that predispose to injury is essential to design effective injury prevention programs.

Endurance running is a sports discipline with an elevated injury incidence due to the high mechanical load produced by weight-bearing and the high running mileage necessary to prepare for endurance competitions [2]. For this reason, a high proportion of injuries in elite endurance runners are muscle or tendon overuse injuries, and the main sites of injury location are lower leg and knee [3,4]. Age, training volume, history of previous injuries, and running kinematics have been considered as the main determinants for the likelihood of suffering endurance running injuries [4,5]. However, genetics might also play a role in the predisposition to injury in some athletes [6], particularly the variations in genes associated with muscle and tendon proteins [7].

α-Actinin-3 is a key component of the skeletal muscle Z-disk in fast-twitch muscle fibers. Hence, it is believed that α-actinin-3 is important for the production of forceful muscle contractions or to resist muscle damage induced by eccentric contractions [8]. A single nucleotide polymorphism (p.R577X; rs1815739) in the *ACTN3* gene (the gene that codifies α-actinin-3) results in the replacement of an arginine (R) with a premature stop codon (X) [9]. Homozygosity for this stop codon (XX genotype) produces an α-actinin-3 deficiency, as opposed to individuals with RX or RR genotypes that express functional α-actinin-3 [10]. Although an α-actinin-3 deficiency is compensated for by a higher expression of α-actinin-2, significant evidence has shown that XX individuals are underrepresented in elite power-oriented athletes, which might be indicative of a negative effect of this genotype on the function of fast-twitch muscle fibers [11].

Recent investigations have found that XX athletes might also be more prone to suffer sports-related muscle injuries when compared to RR counterparts [12,13,14], although this is not always the case [15]. There is greater consensus on the higher predisposition of XX individuals to ankle sprains [16,17,18] and high levels of muscle damage during endurance competitions [19,20]. All this information suggests an a priori predisposition of XX athletes to sports-related injuries, but a lack of replication is present for most current findings that associate genetics with a predisposition to sports injury, as recently suggested [21]. For this reason, we aimed to determine the influence of the *ACTN3* R577X polymorphism on the injury epidemiology of elite endurance athletes, following the methodology of a previous investigation carried out on amateur endurance runners [12]. We obtained information to characterize the influence of this polymorphism on injury epidemiology, such as incidence, conditions, severity, mode of onset, body location, and cause that led to each injury in elite runners with different *ACTN3* genotypes. We hypothesized that elite endurance runners with the XX genotype would have a higher incidence of muscle and ligament injuries when compared to RX and RR athletes.

## 2. Materials and Methods

### 2.1. Participants

Initially, 97 elite Spanish Caucasian endurance athletes volunteered to participate in the study. Participants were cataloged as elite endurance athletes because they competed in national and international events of middle- and long-distance modalities (from 800 m to the marathon). Among them, there were 5 medallists and 20 finalists in International Championships, and 12 champions and 23 medallists in National endurance running competitions. For this sample, two participants were excluded by age (>45 years), and six were excluded because their *ACTN3* genotype was not clearly identified in the genotyping analysis. Age, anthropometric characteristics, running experience, and training status of the final study sample of 89 elite endurance runners are depicted in Table 1. The study protocol conformed to the Declaration of Helsinki for Human Research of 1974 (last modified in 2013) and was approved by the Camilo José Cela University Ethics Committee. Written informed consent was obtained from all participants.

### 2.2. Experimental Design

This investigation was a cross-sectional experiment to determine the effect of the *ACTN3* R577X genotype (RR vs. RX vs. XX) on the injury epidemiology of endurance running-related injuries suffered by elite endurance athletes. For this investigation, participants completed an ad hoc questionnaire between September and November of 2019, seeking to record non-contact injuries sustained during the previous season retrospectively. Only injuries resulting from their training routines or competitions in endurance running activities were recorded. The questionnaire was based on the consensus statement on injury definitions and data collection in epidemiological studies in athletics [22].

### 2.3. Experimental Protocol

In the questionnaire, a recordable injury was defined as a physical complaint or visible damage to any part of the lower limbs sustained by the athlete and assessed by a qualified medical/healthcare practitioner. The injury was recorded irrespective of whether it produced a time loss from training and/or competition or whether it was only a medical attention injury that did not impede normal training. All traumatic injuries, such as the ones caused by a fall or due to contact with an obstacle or another athlete, were discarded as they are not potentially affected by the *ACTN3* genotype. Training exposure was defined as any physical activity conducted under the control or guidance of the coach, with the role of maintaining or improving the athlete’s physical condition. Competition exposure was defined as physical activities conducted in official endurance running competitions. The questionnaire gathered information about the number of injuries sustained in the previous season. Afterward, the information on type, severity, body location, exposure, recurrence, mode of onset, and the possible cause that led to the injury was obtained independently for each recordable injury, following the methodology described by Moreno-Pérez et al. [12]. The questionnaire also gathered information about training routines (endurance running hours per week, resistance training hours per week, number of training weeks per year, number of competitions). Hence, injury incidence was calculated for each athlete as number of injuries per year and as number of injuries per 1000 h of endurance running.

### 2.4. Genetic Testing

Once participants had completed the questionnaire, they were asked to provide two buccal swab samples using self-guided instructions. The samples were stored in envelopes, and genomic DNA was isolated afterward using an organic-based DNA extraction method adapted to Amicon^®^ (Sigma–Aldrich, Madrid, Spain). DNA was extracted within 30 days after sample collection, and the sample was eluted in 50 µL. Positive controls for all genotypes were obtained from the Mexican branch of the CANDELA Consortium. Genotyping of the *ACTN3* rs1815739 polymorphism (c.1858C>T; p.R577X) was conducted using a TaqMan SNP Genotyping Assay (Assay ID: C___590093_1_; Applied Biosystems, Foster City, CA, USA), and the reaction was performed in an Applied Biosystems 7500 Fast Real-Time PCR System (Applied Biosystems).

### 2.5. Statistical Analysis

Data on injury epidemiology were transferred from the questionnaire to an ad hoc database. The normality of each variable was initially tested with the Kolmogorov–Smirnov test and parametric/non-parametric statistics were performed for normally/non-normally distributed variables, respectively. For the continuous variables, genotype comparisons (RR vs. RX vs. XX) were performed using a one-way analysis of variance (ANOVA; followed by Tukey’s post-hoc comparisons) or the Kruskal–Wallis test. For the variables presented as frequency, the differences in distribution among genotypes were identified with crosstabs and χ^2^ tests, including adjusted standardized residuals. Compliance of Hardy–Weinberg Equilibrium (HWE) in the genotype distribution of the sample was tested using χ^2^ tests. Spearman’s rho was used to calculate the association between training volume and the number of injuries per year. All statistical analyses were performed with statistical software (SPSS Statistics 22, IBM Armonk, NY, USA). Descriptive data are presented as means and standard deviations. Statistical significance was set at *p* < 0.05.

## 3. Results

Genotyping for *ACTN3* R577X was successful in 89 out of 95 participants (93.7% of successful genotyping). From the final study sample, 38 (42.7%) athletes had the RR genotype, 35 (39.3%) athletes had the RX genotype, and 16 (18.0%) had the XX genotype (Table 1). The genotype distribution met the HWE. There was no difference in the proportion of men/women elite endurance athletes among genotypes nor in age, anthropometric characteristics, or training variables.

Of the study participants, 32 athletes reported no injury during the preceding season, while the remaining 57 athletes reported a total of 96 injuries. There were no differences in the distribution of athletes with/without injury across the different *ACTN3* genotypes in the whole sample or when analyzing the subsamples of male and female elite endurance athletes (Table 2). The injuries were recorded by a physician (62.1%), by a physiotherapist (32.6%), or by other healthcare providers (5.3%). From the total, 37.9% of injures were diagnosed by magnetic resonance imaging, 28.7% by echography, 4.6% by X-ray, and 28.7% by other clinical testing.

There were no differences in the mean value of injuries per year, nor in the distribution of the number of injuries per year among genotypes (Table 3). However, the median value for injury incidence was higher in RR (3.2, range from 0.8 to 7.4 injuries/1000 h of running) runners than in RX (2.0, range from 0.9 to 7.4 injuries/1000 h of running) and XX (2.2, from 0.9 to 6.3 injuries/1000 h of running; *p* = 0.030) runners. Table 3 depicts epidemiological information about injury conditions in the sample of elite endurance runners. The *ACTN3* genotype did not affect the proportion of time loss/medical attention injuries, the severity of the injury, the distribution of training and competition injuries, the proportion of recurrent injuries, the mode of onset, or the possible cause that led to the injury. However, RR runners had a higher proportion of injuries located in the Achilles tendon, RX runners had a higher proportion of injuries located in the knee, and XX runners had a higher proportion of injuries located in the groin (Table 4, *p* = 0.025). Finally, the *ACTN*3 genotype did not affect the type of injury with a similar distribution of muscle, bone, tendon, and ligament injuries.

## 4. Discussion

In an attempt to increase the scarce knowledge available about the influence of genetics on elite athlete’s susceptibility to injury, we designed a cross-sectional investigation to assess the influence of the *ACTN3* R577X polymorphism on injury epidemiology in elite endurance athletes. This investigation replicates the methods used in a previous study [12], with the only difference being the characteristics of the study sample (elite vs. amateur endurance runners). The main conclusions of this investigation indicate that the *ACTN3* genotype had a slight but interesting influence on injury epidemiology of elite endurance athletes. Specifically, there was a higher injury rate in RR athletes than in RX and XX genotypes, and there were differences in injury location. However, our main hypothesis related to a higher incidence of muscle injuries in XX athletes has not been confirmed with the current data.

The overall injury incidence was 2.8 injuries per 1000 h of endurance running, and 64% of the sample reported at least one injury during the preceding season, which is in agreement with previous data on elite samples [23,24]. Additionally, the number of injuries per year was associated with endurance running volume/year (Spearman’s rho = 0.259; *p* = 0.014), suggesting that a high running mileage was an important injury risk factor in this sample of elite endurance runners as previously found in other types of endurance runners [25,26]. However, the number of injuries per 1000 h of endurance running was 45–60% higher in RR athletes than in RX and XX counterparts. This higher incidence was produced because 21% of the RR athletes sustained three injuries or more (Table 3), while no XX runner sustained ≥ 3 injuries. This is not a unique finding in the literature as a higher injury incidence of RR vs. RX-XX has also been found in amateur runners [12] and in women college athletes [15]. The higher injury incidence among the individuals with a full expression of α-actinin-3 (RR genotype) may be explained by higher levels of muscle strength and a lower range of motion [27,28], which could be translated into more physical stress and lesser joint mobility that ultimately produced an injury.

When taking a close look at injury location, RR had an unusual frequency of injuries located in the Achilles tendon (Table 4), while no XX athlete has reported Achilles tendon injuries in this sample of elite athletes, nor in a sample of amateur runners [12]. In amateur marathoners, XX runners have higher ankle dorsiflexion than RR runners [29]. Interestingly, limited ankle dorsiflexion may increase the risk for mid-portion Achilles tendinopathy in runners [30] and military personnel carrying out a physical training program [31]. Together, all this information suggests that elite RR endurance athletes might be more prone to suffer an overall injury incidence rate, particularly because a high proportion of runners had three or more injuries per year and had an abnormal proportion of Achilles tendon injuries, at least when compared to RX and XX counterparts.

On the contrary, a higher injury incidence of non-contact muscle injuries has been previously reported in amateur athletes [12] and professional football players [13,14]. These investigations indicate that, when isolating muscle-type injuries, XX athletes are 1.8–2.6 times more likely to suffer a non-contact sports injury than RR genotypes. The current investigation does not confirm this outcome because the proportion of muscle strains and muscle ruptures in XX elite athletes was not different from the other two genotypes. The explanation for the lack of higher susceptibility to muscle injuries in our sample of XX elite athletes might be related to their excellent training conditions. In addition to the time devoted to endurance running, this sample of athletes had an average of 148 ± 67 h/year resistance exercise training. The incorporation of resistance training within the physical conditioning program of elite endurance athletes has become more usual in the last few years as this type of training might be effective in enhancing running efficiency but also in improving muscle power and overall performance [32]. Thus, it is possible that the inclusion of a resistance training program at the elite level may have helped to offset the lower strength values found in XX endurance athletes with a low background of resistance training [29]. The current data dispute the notion that catalogues XX athletes as more prone to muscle injuries because research is inconsistent, and the risk of muscle injury according to different *ACTN3* genotypes may be sports-specific. Moreover, it suggests that the potential predisposition of XX sportspeople to muscle-type injury might be overcome by strength training. This is a speculation that merits further investigation.

An interesting outcome of this investigation with elite endurance athletes is the high incidence of groin injuries in XX athletes with respect to the RR and RX genotypes. In the current investigation, one out of three injuries reported by XX runners was located in the groin area, despite this being a body location with a relatively low frequency of injury in endurance runners [24]. The second most common location in XX was the thigh, with a frequency that was double that of RR and RX counterparts. In amateur endurance runners, the knee and the lower leg are the most common body locations for running-related injuries [26,33], particularly in XX runners [12]. During running at low speeds, propulsion is achieved mainly by the structures of the lower leg and soleus, and the gastrocnemius muscles have a particularly important role. At running speeds close to or up to 20 km/h, the capacity of lower leg muscles becomes less relevant, and locomotion is based on the action of the iliopsoas, gluteus maximus, and rectus femoris [34]. In this sample of elite endurance runners, running speeds above 20 km/h are commonly achieved during training and competition, which indicates a high contribution of proximal lower limbs muscles. In this respect, amateur XX runners might be more prone to lower leg injuries, while elite XX runners might be more prone to groin and thigh injuries due to the differences in running speed attained by each group.

The experimental design employed in this investigation presents some limitations that should be addressed to enhance the application of the results. First, the sample size is relatively low, and the extraction of any definitive conclusions about the final association of *ACTN3* genotype and injury epidemiology in elite runners should be made with caution because studies with small sample sizes are more prone to present hidden biases, such as population stratification and cryptic relatedness. Therefore, future investigations in elite runners should be carried out to replicate the results of this study. Additionally, the study sample was composed of elite endurance runners, and the translation of the research outcomes to less trained athletes should be made with caution. This is important as the effect of *ACTN3*XX genotype to increase the incidence of non-contact muscle injury may be higher in amateur than in elite counterparts. Second, the external load of the runners across the year studied, measured as endurance and resistance training volume, was not standardized. Although there were no differences in the number of hours/years devoted to endurance and resistance training among genotype groups (Table 1), future investigations should use experimental designs in which the training and competition load imposed on elite runners is standardized among genotypes. Last, the current investigation only reported recorded injuries that required medical attention and time loss from regular training, while there was no information recorded about complaints that athletes suffered across the year which were not assessed by a qualified medical/healthcare practitioner. Therefore, this characteristic of the experimental design may have biased the results because all severe injuries were recorded, while some of the less severe injuries/complaints may have been unreported. The effect of *ACTN3* genotype on all athletic health problems, irrespective of its consequences on the athlete’s sports participation, or whether the athlete sought medical attention should be investigated, following the recommendations of the International Olympic Committee consensus statement for recording and reporting of epidemiological data on injury in sport [35].

## 5. Conclusions

In summary, the *ACTN3* genotype slightly affected the injury epidemiology of elite endurance athletes with a higher injury rate in RR athletes and subtle differences in injury location. Despite the above-discussed differences, the *ACTN3* genotype did not affect the proportion of time-loss injuries, the severity of the injury, the distribution of training and competition injuries, the proportion of recurrent injuries, the mode of onset, or the possible cause that led to the injury. Regardless of the data obtained in the present study, the evidence linking genetics and injury epidemiology is still emerging, and in the authors’ opinion, it is premature to use genetic testing among elite middle/long-distance runners to effectively predict the risk of injury.

## Figures and Tables

**Table 1:** Age, anthropometric characteristics, running experience, and training status of Spanish elite endurance athletes with different *ACTN3* R577X genotypes.

Variable (units)	RR	RX	XX	*p*-Value
Number (frequency, %)	38 (42.7)	35 (39.3)	16 (18.0)	-
Men (frequency, %)	16 (42.1)	24 (68.6)	8 (50.0)	0.072
Women (frequency, %)	22 (57.9)	11 (31.4)	8 (50.0)	
Age (years)	22.8 ± 4.2	24.5 ± 10.5	26.6 ± 7.0	0.422
≤20 years (frequency, %)	13 (34.2)	11 (31.4)	5 (31.3)	0.078
>20 years ≤ 30 years (frequency, %)	23 (60.5)	21 (60.0)	6 (37.5)	
<30 years (frequency, %)	2 (5.3)	3 (8.6)	5 (31.3)	
Height (cm)	170.2 ± 7.2	172.3 ± 8.4	172.2 ± 6.7	0.353
Body mass (kg)	56.4 ± 8.3	58.1 ± 8.6	57.1 ± 6.3	0.639
Experience (years)	11.5 ± 4.9	12.6 ± 4.9	14.7 ± 6.8	0.098
Endurance running (hours/year)	639 ± 248	650 ± 174	623 ± 237	0.770
Resistance training (hours/year)	152 ± 72	148 ± 67	129 ± 64	0.461
Competitions (number/year)	16.1 ± 6.6	17.5 ± 8.5	16.7 ± 7.1	0.780

Data are mean ± standard deviation (SD) for each genotype.

**Table 2:** Distribution of athletes with/without an injury reported in the preceding season according to their *ACTN3* R577X genotype.

	Variable (units)	RR	RX	XX	*p*-Value
Total	Athletes with injury (frequency, %)	23 (60.5)	24 (68.6)	10 (62.5)	0.766
	Athletes without injury (frequency, %)	15 (39.5)	11 (31.4)	6 (37.5)	
Males	Athletes with injury (frequency, %)	8 (50.0)	19 (79.2)	5 (62.5)	0.153
	Athletes without injury (frequency, %)	8 (50.0)	5 (20.8)	3 (37.5)	
Females	Athletes with injury (frequency, %)	15 (62.8)	5 (45.5)	5 (62.5)	0.449
	Athletes without injury (frequency, %)	7 (31.8)	6 (54.5)	3 (37.5)	

Data are numbers and frequencies (in percentage) of athletes with/without injury reported in the preceding season from the total number of athletes or the number of athletes in each sex.

**Table 3:** Injury incidence, distribution of athletes according to the number of injuries, and distribution of injuries according to severity, exposure, recurrence, mode of onset, and possible cause in elite endurance runners with different *ACTN3* R577X genotypes.

	Variable	All	RR	RX	XX	*p*-Value
Incidence	/per year	1.0	1.0	1.0	1.0	0.321
	/1000 h or running	2.8	3.2	2.0	2.2	0.030
Number	No injury (%)	36.0	39.5	31.4	37.5	0.177
	1 injury (%)	31.5	21.1	40.0	37.5	
	2 injuries (%)	21.3	18.4	22.9	25.0	
	≥3 injuries (%)	11.2	21.1	5.7	0.0	
Time loss	Medical attention (%)	9.2	7.1	10.3	11.8	0.819
	Time loss (%)	90.8	92.9	89.7	88.2	
Severity	Minor (%)	13.4	12.9	12.7	16.0	0.993
	Moderate (%)	51.4	51.6	52.7	48.0	
	Serious (%)	35.2	35.5	34.5	36.0	
Exposure	Training (%)	94.9	95.3	94.9	94.1	0.981
	Competition (%)	5.1	4.7	5.1	5.9	
Recurrence	New onset (%)	61.2	59.5	66.7	52.9	0.598
	Recurrent (%)	38.8	40.5	33.3	47.1	
Mode of onset	Sudden (%)	46.9	45.2	43.6	58.8	0.552
	Gradual (%)	53.1	54.8	56.4	41.2	
Possible cause	Excessive load (%)	62.2	61.9	61.5	64.7	0.526
	Surface (%)	11.2	11.9	15.4	0.0	
	Shoe (%)	8.2	4.8	7.7	17.6	
	Biomechanics (%)	4.1	7.1	2.6	0.0	
	Unknown (%)	14.3	14.3	12.8	17.6	

Data are frequencies (in percentage) from the total of injuries recorded in each genotype.

**Table 4:** Distribution of injuries according to body location and type of injury in elite endurance runners with different *ACTN3* R577X genotypes.

	Variable	All	RR	RX	XX	*p*-Value
Body location	Groin (%)	13.5	4.8 *	13.2	37.5 *	0.025
	Hip (%)	1.0	2.4	0.0	0.0	
	Thigh (%)	15.6	14.3	13.2	25.0	
	Knee (%)	10.4	4.8	21.1 *	0.0	
	Lower leg (%)	15.6	21.4	13.2	6.3	
	Achilles tendon (%)	11.5	19.0 *	7.9	0.0	
	Ankle (%)	9.4	4.8	13.2	12.5	
	Foot (%)	16.7	19.0	13.2	18.8	
	Other (%)	6.3	9.5	5.3	0.0	
Type of injury	Strain/muscle rupture (%)	19.6	22.7	15.0	22.2	0.295
	Stress fracture/other bone injury (%)	13.7	11.4	10.0	27.8	
	Tendinosis/tendinopathy (%)	33.3	31.8	40.0	22.2	
	Sprain/ligament injury (%)	7.8	2.3	12.5	11.1	
	Other (%)	25.5	31.8	22.5	16.7	

Data are frequencies (in percentage) from the total of injuries recorded in each genotype. (*) Different from expected value at *p* < 0.05.

## Data Availability

The data presented in this study are available on request from the corresponding author. The data are not publicly available due to legal restrictions.
